# Burn Injury Induces Proinflammatory Plasma Extracellular Vesicles That Associate with Length of Hospital Stay in Women: CRP and SAA1 as Potential Prognostic Indicators

**DOI:** 10.3390/ijms221810083

**Published:** 2021-09-18

**Authors:** Robert Maile, Micah L. Willis, Laura E. Herring, Alex Prevatte, Cressida Mahung, Bruce Cairns, Shannon Wallet, Leon G. Coleman

**Affiliations:** 1 Curriculum in Toxicology and Environmental Medicine, North Carolina Jaycee Burn Center, Department of Surgery, Microbiology and Immunology, University of North Carolina at Chapel Hill, Chapel Hill, NC 27599, USA; robert_maile@med.unc.edu (R.M.); bruce_cairns@med.unc.edu (B.C.); 2 Curriculum in Toxicology and Environmental Medicine, University of North Carolina at Chapel Hill, Chapel Hill, NC 27599, USA; mlatw@unc.edu; 3 Department of Pharmacology, School of Medicine, UNC Proteomics Core Facility, University of North Carolina at Chapel Hill, Chapel Hill, NC 27599, USA; laura_herring@med.unc.edu (L.E.H.); alex_prevatte@med.unc.edu (A.P.); 4 North Carolina Jaycee Burn Center, Department of Surgery, University of North Carolina at Chapel Hill, Chapel Hill, NC 27599, USA; cmahung@email.unc.edu; 5 Department of Microbiology and Immunology, Division of Oral and Craniofacial Health Sciences, Adams School of Dentistry, University of North Carolina at Chapel Hill, Chapel Hill, NC 27599, USA; Shannon_wallet@unc.edu; 6 Bowles Center for Alcohol Studies, Department of Pharmacology, School of Medicine, University of North Carolina at Chapel Hill, Chapel Hill, NC 27599, USA

**Keywords:** biomarkers, extracellular vesicles, burn injury, trauma, sepsis

## Abstract

Severe burn injury is a devastating form of trauma that results in persistent immune dysfunction with associated morbidity and mortality. The underlying drivers of this immune dysfunction remain elusive, and there are no prognostic markers to identify at-risk patients. Extracellular vesicles (EVs) are emerging as drivers of immune dysfunction as well as biomarkers. We investigated if EVs after burn injury promote macrophage activation and assessed if EV contents can predict length of hospital stay. EVs isolated early from mice that received a 20% total body surface area (TBSA) burn promoted proinflammatory responses in cultured splenic macrophages. Unbiased LC-MS/MS proteomic analysis of early EVs (<72 h post-injury) from mice and humans showed some similarities including enrichment of acute phase response proteins such as CRP and SAA1. Semi-unbiased assessment of early human burn patient EVs found alterations consistent with increased proinflammatory signaling and loss of inhibition of CRP expression. In a sample of 50 patients with large burn injury, EV SAA1 and CRP were correlated with TBSA injury in both sexes and were correlated with length of hospital stay in women. These findings suggest that EVs are drivers of immune responses after burn injury and their content may predict hospital course.

## 1. Introduction

Severe burn injury is a one of the most devastating forms of trauma with over 1.1 million burns each year requiring medical attention in the United States [1,2,3]. Deaths from burn injury are commonly caused by immune-related sequelae such as pneumonia, organ failure and other opportunistic bacterial infections. Paradoxically, these occur days to weeks after repair of the skin barrier function by surgical skin grafting, implicating chronic immune dysfunction. This persistent dysfunction is largely thought to be a result of severe immune dysregulation that occurs early after injury. In fact, burn injury presents as a biphasic systemic immune dysfunction [4]. The acute phase (0–72 h post-injury), also referred to as the burn shock or systemic inflammatory response syndrome (SIRS), results in widespread barrier dysfunction and multiple organ failure. During this immediate-early time period, widespread tissue injury and organ damage results in robust Toll-like receptor (TLR) and cytokine signaling in macrophages (MØ) and neutrophils (NØ) [5,6,7,8]. Following the acute phase, most patients enter a late/chronic phase of immunosuppression (1–2 weeks after burn) referred to as the compensatory anti-inflammatory response syndrome (CARS) phase with an increased susceptibility to infection, which if left uncontrolled can cause organ failure and death [4,9]. Early immune activation is thought to cause persistent maladaptation of immune cells leading to the later CARS phase. Thus, factors that are released early could be used to predict risk for future complications. Though there have been numerous studies to assess the immunological dysfunction associated with burn injury, there have yet to be predictive biomarkers that can be used to assess high-risk patients and their outcomes.

Extracellular vesicles (EVs) have emerged as novel mediators of immune dysfunction across several immune pathologies [10,11,12], including burn injury [5,6,13]. EVs are released from nearly all cell types with their size, concentration, and composition being affected based on pathological conditions. EVs are broadly classified based on their size and biogenesis into three main categories: apoptotic bodies (>1 µm diameter), microvesicles (~0.1–1 µm) and exosomes (~50–100 nm). There is some overlap in size, cargo, and surface markers among the groups, making precise characterization difficult [14,15]. However, exosomes typically originate from the endosomal system while EVs originate mainly from budding of the cellular membrane [10,14]. We previously reported that EVs from burn-injury patients contain DAMPs such as HMBG1 that are known to promote sepsis and organ failure [5,16]. Others have reported that EV numbers after burn are associated with sepsis and clinical severity and that EVs are bioactive [13,17]. In this work, we investigated whether cargo in EVs isolated early after burn injury is altered and associated with hospital course. We hypothesized that plasma EVs released early after burn injury promote proinflammatory activation of peripheral macrophages and that specific cargo could be identified in these EVs to serve as a biomarker to identify at-risk patients.

**Figure 1:**
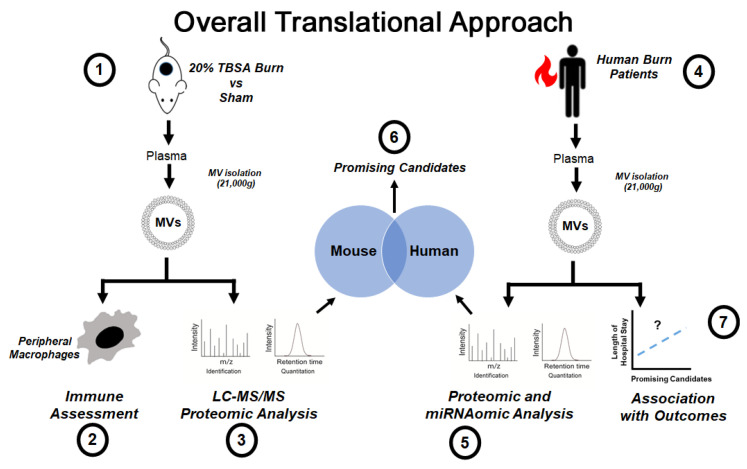
Overall Translational Experimental Design. (1) Mice undergo a 20% total body surface area (TBSA) burn injury, and EVs isolated within 72 h after injury. (2) Mouse burn EVs are tested for pro-inflammatory capacity in mouse splenic macrophages. (3) Mouse burn EVs are assessed for protein content using LC-MS/MS proteomic analysis. (4) EVs are isolated from human burn patients with severe burn injury. (5) Proteomic and miRNAomic analysis of human EVs is performed. (6) Proteomic profiles of human and mouse burn EVs are compared, and promising candidates identified. (7) Promising candidates were measured in a larger cohort of human burn patients and associated with length of hospital stay.

In order to test this hypothesis, we used a translational approach (Figure 1). First, we used our preclinical large burn model to test the effect of early post-burn plasma EVs on peripheral splenic macrophages. We then used unbiased proteomic assessments to identify molecular candidates that could be assessed in human burn patients. Next, we moved to human patient samples from the UNC Jaycee Burn Center and performed unbiased proteomic and miRNAomic assessments of plasma EVs from human burn patients to compare their cargo with those found in the mouse. Third, we measured the levels of candidate markers identified from our unbiased assessments in a larger cohort of human burn patients and determined their relationship on patient outcomes, specifically length of hospital stay. This translational approach allowed us to identify two potential biomarkers that have immunomodulatory abilities found in EVs early after burn injury– serum amyloid A1 (SAA1) and C-reactive protein (CRP).

## 2. Results

### 2.1. Plasma EVs from Burn Mice Reproduce Burn-Associated Immune Dysfunction in Cultured Splenic Macrophages

**Figure 2:**
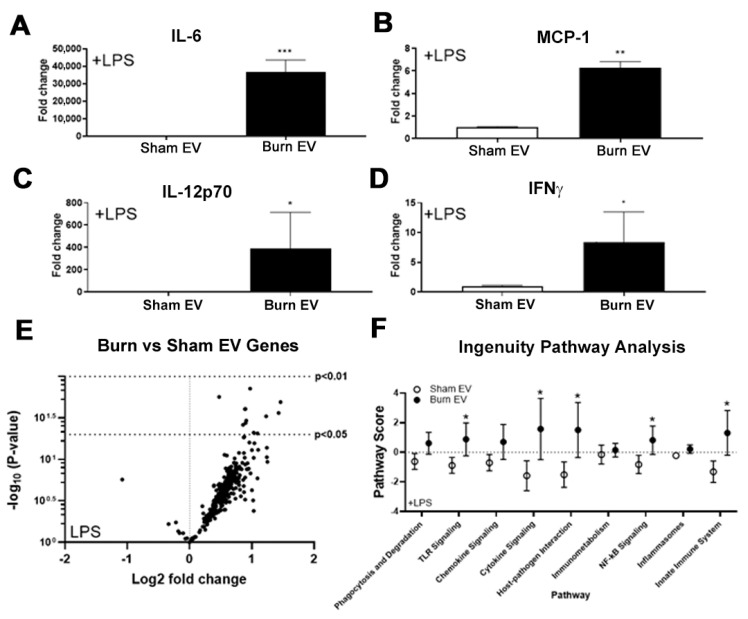
Plasma extracellular vesicles (EVs) after burn injury promote pro-inflammatory signaling in macrophages. Adult mice underwent a 20% total body surface area (TBSA) burn injury and plasma EVs were collected 48 h after injury. EVs from burn injury mice (Burn EV) or sham injured mice (Sham EV) were applied (3 × 10^7^/well) to splenic macrophages +/− LPS (100 ng/mL) for 24 h. (**A**–**D**) Media cytokines were measured by multiplex. Burn EVs cause robust increases in pro-inflammatory (**A**) IL-6, (**B**) MCP-1, (**C**) IL-12p70, and (**D**) IFN¦Ã in macrophage media. * *p* < 0.05, ** *p* < 0.01, *** *p* < 0.001 vs. sham EVs, *t*-test. (**E**,**F**) Macrophage lysates were harvested, mRNA isolated, and gene changes measured by NanoString. (**E**) Volcano plot of several genes significantly induced by burn-EVs relative to sham EVs. (**F**) nSolver pathway analysis scores of changes in measured immune pathways. Burn EVs significantly altered TLR signaling, cytokine signaling, host-pathogen interactions, NFkB signaling, and the innate immune system in macrophages relative to sham EVs.

A hallmark of burn injury is profound immune dysregulation, with macrophages being a key compartment impacted [4,5,7,8,18,19], ultimately resulting in susceptibility to infection and mortality. Therefore, we hypothesized burn-derived EVs would significantly alter macrophage responses. Mice underwent a 20% TBSA cutaneous burn injury (*n* = 6 per group) or sham injury and plasma EVs were isolated three days after injury. EV concentration was similar for EV preparations for each group with diameters between 60–400 µm as measured by nanoparticle tracking analysis (NTA, not shown). EVs from sham or burned mice (sham EV or burn EV, respectively, 3 × 10^7^ per well) were added to primary murine splenic macrophage cultures (1 × 10^6^ cells/well). To define the impact of burn EVs on macrophage function, EVs were added in the presence of LPS, a well-defined pro-inflammatory Gram-negative bacteria-associated molecule. This models the immune environment seen shortly after burn injury where levels of bacterial pathogens are increased due to loss of skin barrier function. After 24 h of culture, supernatant was assessed for cytokine and chemokine levels by multiplex bead-based ELISA analysis and purified RNA from cell lysates for NanoString multiplex immune gene expression analysis. Compared to sham, burn EVs robustly induced secretion of IL-6 (35-fold, *** *p* < 0.001, Figure 2A), MCP-1 (6-fold, ** *p* < 0.01, Figure 2B), IL-12p70 (400-fold, * *p* < 0.05, Figure 2C) and IFNγ (7-fold, * *p* < 0.05, Figure 2D). These cytokines are also found to be increased peripherally early (~1–3 days) after burn injury in human burn patients and mouse models [4,5,7,8,19,20,21,22,23,24,25,26,27].

For cellular gene expression, we utilized the commercially available Mouse Immunology Panel v3.0 which allows for 561 mRNA general immune genes to be quantified simultaneously. The volcano plot in Figure 2E demonstrates the change in gene expression along with its associated significance, following macrophage exposure to burn EV versus sham EV. Significantly altered genes (*p* < 0.01) are presented in Supplemental Table S1 (data also uploaded to NCBI Gene Expression Omnibus GEO). Principal components analysis (PCA) of this gene expression dataset derived pathway scores (PS), based on the individual gene expression levels for all the measured genes within a specific pathway [28], to identify pathways active in the macrophages. Generally, positive PS indicate pathways which are highly affected based on gene expression patterns observed, while negative PS indicate pathways which are not as affected based on gene expression patterns observed. Figure 2F shows the PS driven by EV derived from sham or burn injury. Pathways shown are predicted with *p* < 0.05 confidence based on causal gene expression. PS driven by EV after burn injury are generally positive for many key immunological pathways including TLR signaling, phagocytosis, chemokine signaling, etc., compared to the generally negative PS after exposure to sham injury-derived EV.

### 2.2. Large Burn Injury in Mice Alters Plasma EV Proteomic Profile

**Figure 3:**
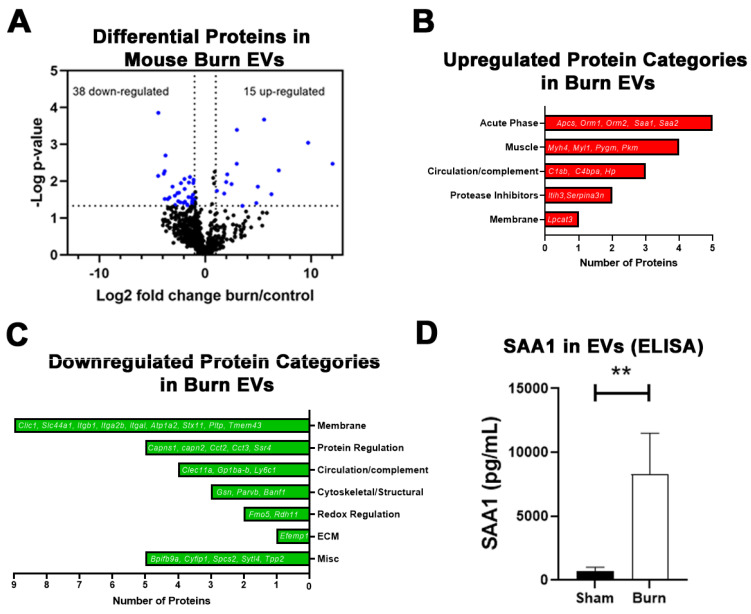
Proteomic changes in mouse plasma EVs after burn injury. Adult mice underwent a 20% total body surface area (TBSA) burn injury and plasma EVs were collected 72 h after injury and protein content measured by LC-MS/MS. (**A**) Differentially expressed protein peptides in burn vs. sham EVs (LFQ ratio, *p*-value Student’s *t*-test, *n* = 3 per group). (**B**) Characteristics of proteins that are increased in burn EVs relative to controls. (**C**) Characteristics of proteins that are decreased in burn EVs relative to controls. (**D**) ELISA measurement of SAA1 in plasma EVs from sham and burn mice. SAA1 protein was robustly increased in burn-EVs compared to sham. ** *p <* 0.01, *t*-test, *n* = 4 sham, 8 burn.

Since we found that burn EVs cause significant dysregulation of immune function ex vivo, we began to assess EV protein content. Again, mice underwent a 20% TBSA burn followed by LC-MS/MS on plasma EVs. EVs primarily in the microvesicle (MV) size range (0.1–1 µm in diameter) were isolated by centrifugation as we have previously reported [5,29,30]. LC-MS/MS identified over 700 proteins that were quantifiable, with 53 proteins showing differential levels between burn and control EVs (Table 1). Fifteen proteins were increased in EVs from burned mice compared to 38 proteins that were downregulated after burn injury (Figure 3A). Upregulated proteins fell into five main categories: acute phase proteins (five detected), muscle proteins (four), circulatory/complement proteins (three), protease inhibitors (two) and membrane proteins (one) (Figure 3B). Of the 38 downregulated proteins, the majority were membrane proteins (nine), followed by protein regulators (e.g., proteases, chaperones, five), circulatory/complement proteins (four), cytoskeletal/structural proteins (three), redox reaction regulatory proteins (two), extracellular matrix proteins (ECM, one), and five proteins whose function has not yet been clearly defined (Figure 3C). The largest measured increase was in SAA1 (~10-fold), an acute phase protein that can modulate innate immune function as a ligand for TLRs. To confirm the LC-MS/MS-detected increase in SAA1, we performed an ELISA which found a comparable 11.5-fold increase in SAA1 in EVs (Figure 3D). A non-significant trend toward a two-fold increase in SAA1 protein in MV-depleted plasma was detected (*p* = 0.11, data not shown), indicating the greatest increase in SAA1 protein is within the MV compartment.

### 2.3. Burn Injury in Humans Alters Proteomic Profile of Plasma EVs with Some Similarities to Mice

**Figure 4:**
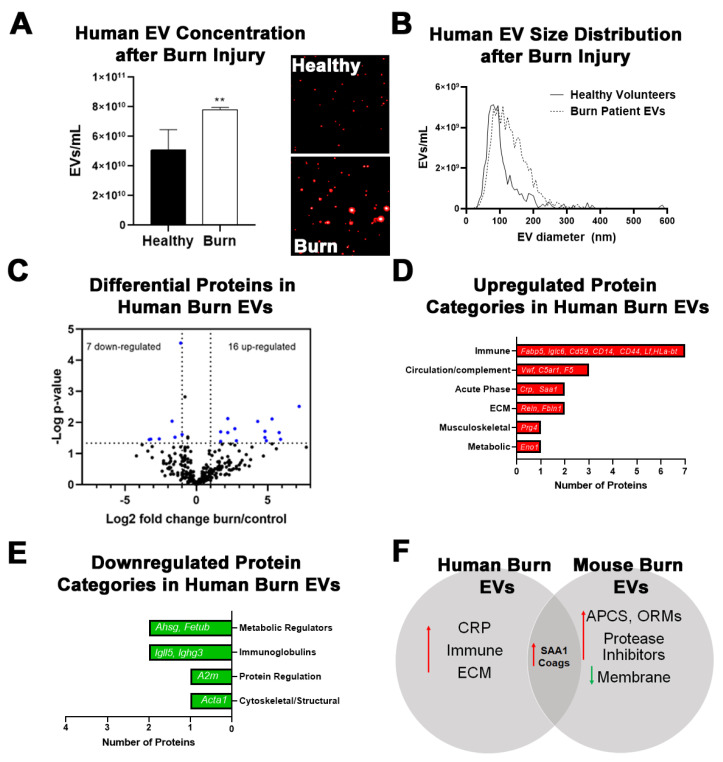
Proteomic changes in human burn patient plasma EVs. Plasma EVs from human burn patients with severe burn injury were isolated within 72 h of admission. Protein content was measured by LC-MS/MS. (**A**) EV number was measured by NTA. Human burn patients showed an increase in circulating plasma EVs compared to healthy donors. ** *p <* 0.01, *n* = 3/group. Inset shows representative images of EVs captured by the ParticleMetrix^TM^ machine. (**B**) NTA size analysis shows the majority of EVs are in the 100–400 µm diameter size range, with burn EVs showing increased numbers in the MV size range. (**C**) Differentially expressed protein peptides in burn vs. healthy donor EVs (LFQ ratio, *p*-value Student’s *t*-test, *n* = 3 per group). (**D**) Characteristics of proteins that are increased in burn EVs relative to healthy individuals. (**E**) Characteristics of proteins that are decreased in burn EVs relative to healthy individuals. (**F**) Comparison of robust changes between human burn and mouse burn EVs. Both SAA1 and coagulation proteins were increased across species.

Since several protein changes were found in burn EVs after injury in mice, we performed LC-MS/MS proteomic analysis on plasma EVs isolated from human burn injury patients (>20% TBSA) within 72 h of injury. NTA using the Particle Metrix platform found a 52% increase in plasma EVs in the human burn patients compared to healthy donor controls (Figure 4A). The Particle Metrix machine was able to visualize this increase (Figure 4A, inset). Analysis of the size distributions found the isolated EVs to be primarily in the size range of 100–400 µm diameter (Figure 4B), with increased numbers of EVs in this size range in the human burn patients. Using LC-MS/MS, a total of 301 specific proteins were identified, with 23 proteins differentially regulated in burn-injured patients versus healthy controls (Table 2). Sixteen proteins were significantly upregulated while 7 showed reduced expression (Figure 4C). Of the upregulated proteins, the majority were immune-regulating proteins including Fabp5, Iglc6, CD59, CD14, CD44, Lft, and HLA-B (Figure 4D). Three proteins associated with the complement system (Vwf, C5ar1, and F5) were also increased. The largest fold-change increases, similar to findings in the mouse, were in acute phase response proteins CRP and SAA1. Extracellular matrix (ECM) proteins (Reln, Fbln1) as well as musculoskeletal proteins (Prg4) and metabolic regulators (Eno1), were the remaining increased proteins. Downregulated proteins include metabolic regulators (Ahsg and Fetub), immunoglobulins (Igll5 and Ighg3), and cytoskeletal (Acta1) proteins (Figure 4E). There were some systems level similarities between the human burn EVs and the mouse burn model. Particularly, both groups showed robust increases in the acute phase response (APR) protein serum amyloid A-1 (Saa1) as well as an increase in complement cascade proteins (Figure 4F). Notable differences between the two species include a more apparent increase in immune cell-related and extracellular matrix proteins in the humans, while mice showed induction of additional APR proteins and protein inhibitors with a reduction of multiple membrane-associated proteins and protein regulators. Remarkably, although significantly more proteins were measured in the mouse EVs than in humans, a similar percentage of genes was significantly changed in both species (~7%). Mice are far more resilient than humans in their recovery from a severe burn injury. Thus, inter-species differences may provide insights into protective factors. Common changes across both species, however, may provide insight into shared mechanistic pathways involved in the response to burn injury. Since EVs carry diverse protein and nucleic acid cargo, we next assessed changes in circulating miRNAs in EVs from human burn patients.

### 2.4. Burn Injury in Humans Alters miRNAs in Plasma EVs

**Figure 5:**
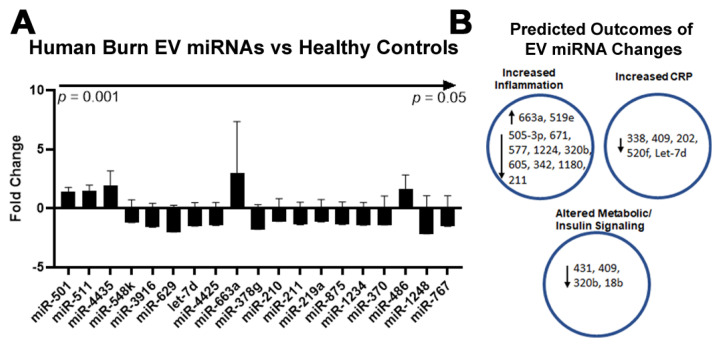
Proinflammatory miRNA signature in EVs from human burn patients by NanoString. Plasma EVs were isolated from human burn injury patients during the first 48 h of admission or healthy controls and miRNA content assessed by NanoString. (**A**) NanoString assessment of human burn patient EVs revealed that 26 miRNAs were significantly altered, 22 of which showed reduced expression. *n* = 3 per group. (**B**) Predicted outcomes of changes in differentially expressed miRNAs. Directional changes in 11 of the miRNAs would promote inflammation, while reductions in 5 miRNAs that target CRP would promote CRP expression, and reductions in 4 miRNAs that target metabolic and insulin signaling could alter cellular metabolism.

In addition to their protein cargo, EVs contain a diverse profile of miRNAs that have been used in a various disease settings such as pancreatic cancer in attempts to identify potential biomarkers [31]. Therefore, we isolated miRNAs in plasma EVs from the same patients that were assessed for protein changes by LC-MS/MS using the human miRNA panel v2. The expression of 800 miRNAs relative to healthy control subjects was measured. This analysis identified 26 miRNAs (Figure 5A) that were differentially expressed, four of which showed increased expression (miR-663a, miR-363, miR-4435, and miR519e) while 22 miRNAs showed reduced expression compared to healthy controls (miR-505, miR-671, miR-151b, miR-577, miR-431, miR-409, miR-1224, miR-575, miR-4425, miR-320b, miR-202, miR-338, miR-605, miR-520f, miR-342, miR-651, miR-1180, miR-211, let-7d, miR-1910, miR-18b, and miR-1197). These miRNAs have several known functions. By performing a comprehensive literature search (Table S1) as well as prediction analysis of miRNA targets using TargetScan, we found three main relevant regulatory categories were altered (Figure 5B), including the regulation of inflammation (11 miRNAs), regulation of the APR protein CRP (five miRNAs), and regulation of metabolism (four miRNAs). Interestingly, the directionality of the changes in miRNA was consistent with expected systems level functional changes seen with burn injury. For instance, the expression of several miRNAs that are known to limit proinflammatory responses was reduced (miR 505-3p, miR 671, miR 577, miR 1224, miR 320b, miR 605, miR 342, miR 1180, and miR 211). However, five miRNAs previously found to lower the expression of CRP, were found to be increased. Thus, EV miRNAs might have functional roles in regulating burn-associated protein changes and may also be used as proxies for identifying altered biological systems.

### 2.5. Plasma EV Number Correlates with Disease Severity

Since we found a potential role for plasma EVs in modulating immune responses post-burn injury in the pre-clinical mouse model, and we found robust protein changes in APR proteins CRP and SAA1, we assessed plasma EV composition in a larger cohort of human burn patients from our human burn patient repository. EVs isolated early after burn injury from 50 burn patients were isolated. The clinical characteristics of these patients was similar to our mouse burn injury model with an average %TBSA injury of 18.4% and plasma collected within the first 72 h after injury (Table 3). The majority of patients were male (68%) consistent with historic trends in burn patient populations.

**Figure 6:**
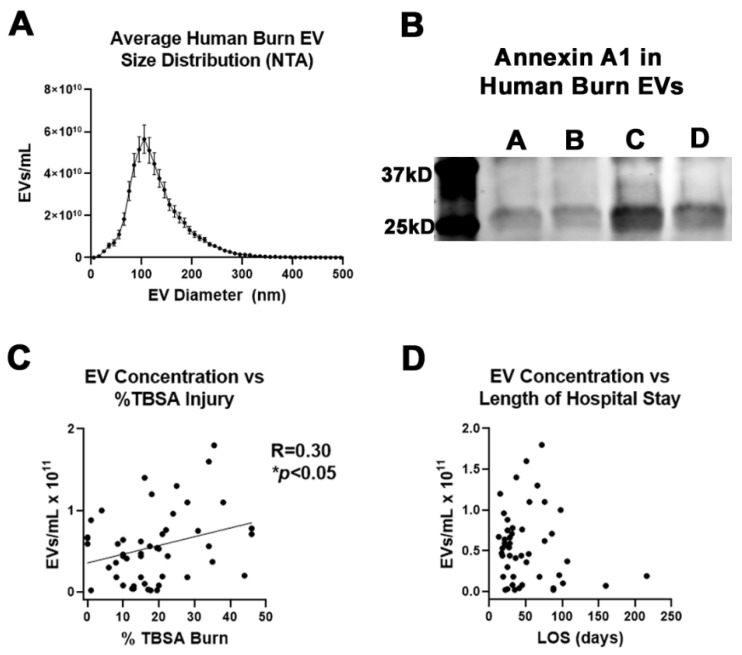
Assessment of human plasma EVs from human burn patients. Plasma EVs were isolated from 50 human burn patients within the first 72 h of admission. (**A**) Assessment of EV sign distribution by nanoparticle tracking analysis (NTA) found the majority of EVs were within the MV size range. (**B**) Western blot on EVs isolated from 4 human burn patients stained for MV marker Annexin-A1. (**C**) EV concentration was positively correlated with size of burn injury. (**D**) No correlation between EV concentration and length of hospital stay was found across subjects.

We first measured the number of EVs from each subject in plasma collected within 72 h of burn injury. This early time point was assessed in hopes of identifying mediators that might predict outcome in this patient population. The average concentration of EVs was 5.4 × 10^11^/mL (Figure 6A). There was no difference in EV concentrations between males and females, and the majority of vesicles (>70%) were within the MV size range of 100–500 nm. To confirm the presence of MVs, Western blot was performed on human EVs for MV marker Annexin-A1 (Figure 6B) [14]. Each of the subjects assessed was found to have Annexin-A1 in the isolated EVs, consistent with the presence of MVs in the preparation. The concentration of EVs was found to be positively correlated with the %TBSA burn injury (r = 0.3, * *p* < 0.05, Figure 6C). However, there was no association between plasma MV concentration and the length of stay (LOS) in the hospital, indicating EV number alone early after burn injury is not a strong predictor of hospital course. Therefore, we turned our attention to CRP and SAA1, which were found to be increased by LC-MS/MS in the smaller subset of patients above.

### 2.6. Human Burn Patients Have Increased Levels of CRP and SAA1 in Plasma EVs That Predict Hospital Length of Stay

**Figure 7:**
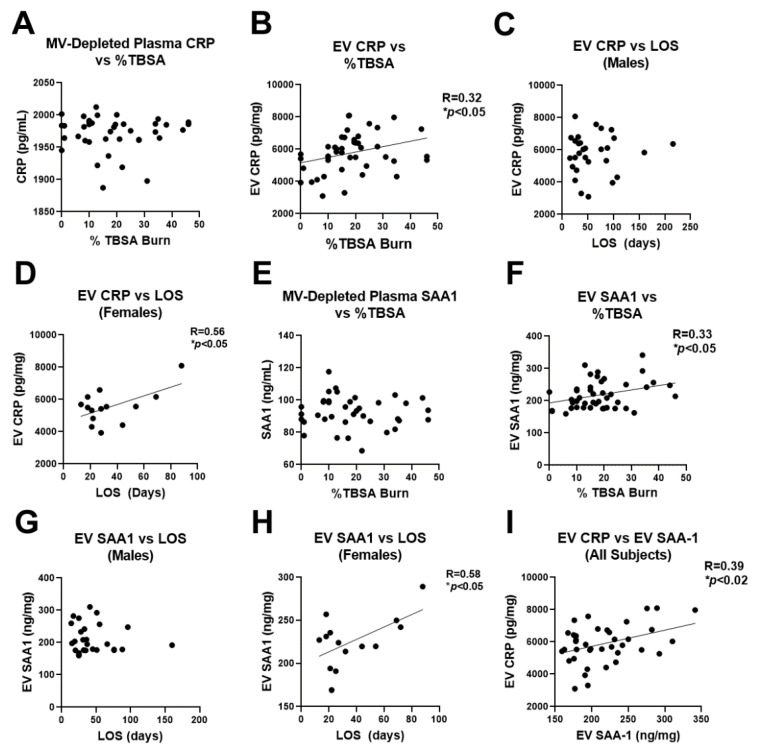
Association of EV CRP and SAA-1 in EVs with length of hospital stay in severe burn injury. EVs were isolated from 50 human burn patients and levels of CRP (**A**–**D**) and SAA1 (**E**–**H**) measured by ELISA and their association with length of stay (LOS) in the hospital were determined. (**A**) CRP levels from MV-depleted plasma showed no association with severity of burn injury. (**B**) CRP levels in EVs from human burn patients showed a positive correlation with severity of burn injury. r = 0.32, * *p* < 0.05. (**C**) CRP levels in EVs of male human burn patients was not related to LOS. (**D**) CRP concentration in EVs from female burn patients was positively correlated with hospital LOS (r = 0.56, * *p* < 0.05). (**E**) SAA1 protein concentration in MV-depleted plasma showed no association with severity of burn injury. (**F**) SAA1 concentrations in EVs from human burn patients showed a positive correlation with severity of burn injury. r = 0.33, * *p* < 0.05. (**G**) SAA1 concentrations in EVs of male human burn patients was not related to LOS. (**H**) SAA1 concentration in EVs from female burn patients was positively correlated with hospital LOS (r = 0.58, * *p* < 0.05). (**I**) Concentrations of CRP and SAA1 in EVs were significantly correlated with each other. r = 0.39, * *p* < 0.02.

Since both CRP and SAA1 were found to be increased in human burn EVs isolated during the first 72 h of injury (with SAA1 also being increased in mouse burn-EVs), we measured CRP and SAA1 by ELISA in EVs and the remaining plasma supernatant in our larger cohort to assess for their association with burn injury severity and LOS in the hospital. LOS is a key clinical endpoint that conveys the severity of the illness or disease. This would allow us to determine if these identified markers could serve as biomarkers to identify high-risk patients. As shown in Figure 6A, the majority of isolated EVs were in the MV size range (100–500 nm diameter). However, since some overlap occurs between exosomes and MVs near the 100 nm size range, we refer to the plasma supernatant as MV-depleted plasma (as apoptotic bodies and MVs are primarily removed by the 21,000× *g* spin, yet exosomes will remain) and the 21,000× *g* pellet as EVs since there may be both MVs and larger exosomes present. In the MV-depleted plasma, CRP levels were stable across all severities of burn injury (Figure 7A) and LOS (not shown); however, in the EV fraction, CRP levels were positively correlated with the severity of burn injury (Figure 7B). In males, the level of CRP in EVs isolated during the first 72 h of admission did not associate with hospital LOS (Figure 7C). However, in females, EV CRP levels were strongly associated with LOS (r = 0.56, * *p* < 0.05, Figure 7D). A similar pattern was found with SAA1. There was no association of SAA1 in the MV-depleted plasma with burn severity (Figure 7E); however, EV SAA1 was positively correlated with the size of burn injury (Figure 7F). There was no correlation between EV SAA1 and LOS in males (Figure 7G); however in females, EV SAA1 was strongly correlated with LOS (r = 0.58, * *p* < 0.05, Figure 7H). Both CRP and SAA1 levels were correlated with each other (Figure 7I), suggesting similar pathways may result in their secretion in vesicles. Together, these data implicate these two APR proteins that were identified by unbiased proteomic analysis as early indicators of burn injury severity that are associated with length of hospital stay in females.

## 3. Discussion

Burn injury is one of the most severe forms of trauma and often results in profound immune, metabolic, and cardiovascular dysregulation. Even long after the repair of skin barrier function by skin grafting, persistent physiological derangements persist. After resolution of the initial burn shock, mortality occurs weeks after injury, mostly due to infectious or cardiovascular complications [32,33,34,35,36,37,38,39]. The ability to identify high-risk individuals at the time of injury could greatly benefit care. Further, the identification of molecular mediators that could be targeted for intervention could lead to new therapeutic approaches. Currently, the standard of care for severe burn injuries is primarily supportive care and restoration of skin barrier function. This involves volume resuscitation during the early shock phase followed with critical ICU care providing respiratory and other organ support as needed. Once recovery from skin grafting or other surgical interventions is complete, normalization of physiology is the priority. Unfortunately, many patients experience secondary infections days to weeks after skin coverage is regained along with cardiopulmonary and other complications. Currently, there exist neither any early interventions that can prevent future morbidity nor any early diagnostic approaches to identify at-risk individuals.

In this study, we investigated if circulating EVs that are released early after burn injury promote immune dysfunction and could be used as biomarkers to identify at-risk patients. Prior work has found that EVs are increased after burn injuries in human patients and rodent models, and carry proinflammatory cytokines and TLR-activating DAMPs [5,13]. Here, we found that EVs secreted during the first 48 h after burn injury in mice are immunogenic, profoundly increasing proinflammatory cytokine secretion and proinflammatory signaling in splenic macrophages. TLR, cytokine, host-pathogen interaction, NFκB and innate immune system pathways were strongly increased by burn EVs. These findings are consistent with a recent report using RAW264.7 cells and bone marrow-derived macrophages [6]. These pathways are key for innate immune function and are known to play a role in burn-induced immune responses [40,41], suggesting EVs might play a role in driving immune responses early after burn injury. Importantly, we recently reported that post-burn EVs have similar effects in vivo as our findings here in vitro [6]. It is currently unclear if these responses might be protective, detrimental, or both. However, the majority of work suggests that early proinflammatory responses after burn injury may ultimately be counter-productive, resulting in long-term immune dysfunction after the resolution of the early burn shock [42,43]. Therefore, finding therapeutic targets that either reduce or modulate heightened early proinflammatory responses could be beneficial for patient care.

In order to identify new potential mediators in EVs that could be targeted therapeutically, we performed LC-MS/MS and NanoString^TM^ analysis on burn EVs. We found some commonalities between the components in both human and mouse EVs, such as the APR protein SAA1 and increases in circulating complement/coagulation factors. These differences were found with sample size of 3 per group in each species. If the sample size were increased, it is possible that additional mediators would reach statistical significance, making this a limitation of this study. The finding of increased C5aR1 in human burn patients is consistent with a recent report finding increased C5aR1 on granulocyte derived MVs in polytraumatized patients that could promote coagulation [44]. Further, we found CRP was increased in human burn patient EVs. CRP binds to phosphatidylcholine in membranes of apoptotic or necrotic cellular remains, or EVs potentially, and activates complement cascades to promote their uptake by phagocytic cells [45,46,47]. After burn injury, a significant amount of necrotic and apoptotic debris is released that may warrant this induction of CRP in EVs. SAA1 also promotes phagocytosis in macrophages and is a ligand for TLR4 [48,49,50], which is induced early after burn injury [7,20]. Both SAA1 and CRP correlated with %TBSA burn injury across all subjects and length of hospital stay in females. It is currently unclear why there were differences between males and females. However, immune regulation between the two sexes is often divergent in the setting of injury [9]. Similar to our findings, CRP was recently reported to be increased in EVs of patients with sepsis and to promote proinflammatory responses in macrophages [51].

We found that neither CRP nor SAA1 in EV-depleted plasma was associated with severity of burn injury or length of hospital stay. This suggests that EVs may be a specific and active reservoir that can regulate post-traumatic pathology and be assessed for biomarkers. It is important to note that we used a centrifugation method to isolate EVs, which could readily be adopted in most hospital laboratories. It is difficult to definitively determine the type of EV present in any preparation, as size overlap between exosomes and MVs occurs in the size range of these preparations [14,15]. Additionally, it is possible that larger lipoprotein complexes could be included. However, our protocol mimics the most likely procedures that could be feasibly used by most hospital laboratories. Regardless of the exact subcellular vesicular localization, we identify relevant molecules that may serve as potential biomarkers or therapeutic targets.

Our findings, as well as other work, suggest that EVs are specific mediators of signaling after trauma that might be targeted therapeutically or used as predictors of hospital course. Our miRNA assessments and ex vivo EV transfers further suggest that early proinflammatory responses seen after burn injury are at least in part initiated by EVs. For instance, in human burn EVs, several miRNAs that are known to or are predicted to dampen inflammatory signaling were reduced, while some that promote proinflammatory signaling pathways were increased (see Figure 5 and Table S1). Further supporting a role for EVs as dynamic regulators of molecular signaling was the reduction in the presence of five miRNAs known to inhibit CRP expression, while CRP itself was increased in human burn EVs. Coupled with the functional studies in adoptive transfer to splenic macrophages, this work suggests that EVs are actively regulating immune and potentially other responses after severe trauma. We recently reported that EVs isolated from burn-injured mice and transferred to naïve, unburned mice recapitulate much of the immune response seen with burn injury itself [6]. Further, in their study finding increased EV CRP in sepsis patients, Fendl et al. found that depletion of CRP+ EVs from septic patients reduced the pro-inflammatory activity of septic plasma. This occurred even though the majority of CPR in plasma was not bound to EVs [51]. Therefore, this and other work implicates EVs as specific mediators of immune activation after trauma. Further work is needed to clearly define the temporal changes in EV contents and activity after injury. Additionally, studies assessing the effects of human post-burn EVs on human immune cell function are needed. In future studies we plan to do this using induced pluripotent stem cells (IPSCs) differentiated into human monocytes, neutrophils, epithelial cells, and endothelial cells.

Together, findings presented here as well as recent work by other groups indicate that EVs are key regulatory mediators in settings of trauma. Their contents may predict outcomes in specific patient populations such as males versus females. They also provide insight into cellular and organ dysfunction throughout the body and may be used to identify novel therapeutic targets or approaches. Future work will study the utility of EV replacement therapy or dialysis in improving outcomes in the setting of severe burn injury.

## 4. Materials and Methods

### 4.1. Mouse 20% TBSA Thermal Injury

All procedures were conducted in strict adherence to the Guide for the Care and Use of Laboratory Animals of the National Institute of Health and approved by the University of North Carolina Institutional Animal Care and Use Committee under protocol #21-082. Mice were housed in American Association for Accreditation of Laboratory Animal Care (AAALAC)-accredited facilities with 24 h veterinary care and close observation throughout the experiment. Measures were taken to alleviate suffering, with all injuries performed under avertin anesthesia with appropriate analgesia. Mice underwent a 20% total body surface area (TBSA) thermal injury to model a large burn injury in humans as described previously [5,6,22,23]. Briefly, C57BL6 mice (female, 6–8 weeks old, 15–20 g) first were anesthetized with tribromoethanol/avertin (475 mg/kg; Sigma-Aldrich, Burlington MA, USA). Their targeted region on the dorsum was then shaved (NC0854145; Fisher, Pittsburg, PA, USA) prior to injection of subcutaneous morphine sulfate (3 mg/kg; Westward, Berkeley Heights, NJ, USA). Four defined skin locations were then contacted for 10 s with a copper rod heated to 100 °C in a water bath. Mice were then resuscitated with Ringer’s lactate (0.1 mL/g body weight). Post-procedure analgesia was maintained with morphine sulfate-supplemented water (60 μg/20 g mouse) ad lib for the duration of the experiment. Sham mice underwent identical treatment minus application of a heated copper rod. Mice were monitored at least twice daily for the duration of the experiment. Mice that showed signs of distress including a >15% loss of body weight, difficulty breathing, hunching over, dehydration, inactivity, or growing lesions were humanely euthanized immediately. There was zero burn-related mortality.

### 4.2. EV Isolation, Quantification, and Sizing

EVs were isolated from plasma of human burn patients collected 0–72 h after injury or mice at 24 and 72 h after injury using sequential centrifugation as we have described previously. This process results in isolation of EVs primarily between 100 nm and 1 μm in diameter (primarily the MV size range), which we have confirmed by flow cytometry and nanoparticle tracking analysis (NTA) [5,29,30]. Briefly, anticoagulated plasma was centrifuged at 2000× *g* for 20 min to remove cells, followed by a 10,000× *g* spin of the supernatant for 30 min to remove cellular debris, and a final 21,000× *g* spin of remaining supernatant for 1 h to pellet the EVs. EV concentration (EVs/mL) and size distributions were measured by NTA using the ZetaView QUATT Particle Metrix machine at a 1:1000 dilution in ultrafiltered PBS.

### 4.3. Splenic Macrophage Culture and EV-Stimulation

Total splenocytes, which were obtained from spleen female C57BL/6 mice, underwent red-cell lysis by Ammonium-Chloride-Potassium (ACK) buffer exposure, were suspended into RPMI1640 media supplemented with 10% fetal bovine serum, 1% penicillin/streptomycin, and 100 ng/mL rhM-CSF at a density of 3 × 10^5^ cells/mL in 75 mL tissue culture plates, and cultured at 37 °C and 5% CO_2_ for 7 days. Non-adherent cells were removed every other day. Resultant adherent splenic macrophages were harvested from plates by dispase treatment, and 1 × 10^6^ were replated into 24 well plates and rested for 24 h. Cells were then exposed to 3 × 10^7^ EVs from burn- or sham-injured mice for 24 h. Supernatants and cells were harvested for analyses.

*EV adoptive transfer to splenic macrophage cultures:* EVs were collected from plasma of six mice 24 h after burn injury (burn-EVs) or sham exposure (sham-EVs). 1 × 10^10^ EVs in sterile PBS were adoptively transferred to 1 × 10^6^ splenic macrophages, which was ~15% of total media EVs of naïve splenic macrophage cultures. Treated cells were harvested 24 h after exposure for cytokine and chemokine measurement.

### 4.4. Measurement of Cytokines and Chemokines in Burn- and Sham-EV Treated Splenic Macrophages

Macrophage media and cell lysates were collected, and the Bio-Plex Multi-Plex Immunoassays (BioRad) used to measure IL-1β, IL-2, IL-6, IL-8, IL-10, IL-12(p70), IFN-g, MCP-1, and TNFα, according to the manufacturer’s instructions. The Bio-plex 200^®^ system with Bio-Plex Manager and Bio-Plex Data Pro Software was used to capture and analyze data. A 5-paramater logistic spline-curve fitting method was used to calculate final values which were measured in pg/mL.

### 4.5. Macrophage Immune Gene Detection and Quantification by NanoString

Isolation of mRNA was performed as previously described [5]. Briefly, macrophages were lysed with TRIZOL buffer (Sigma) and total RNA was isolated by chloroform extraction and quantified (A_260_/A_280_ and A_260_/A_230_) using a nanodrop 2000 TM spectrophotometer (NanoDrop Technologies, Waltham, MA, USA). NanoString technology and the nCounter Mouse Immunology Panel (Nanostring Technologies, Seattle, WA, USA) was used to simultaneously evaluate 561 mRNAs in each sample [52]. Each sample was run in triplicate. Briefly, a total of 100 ng mRNA was hybridized to report-capture probe pairs (CodeSets) at 65 °C for 18 h. After this solution-phase hybridization, the nCounter Prep Station was used to remove excess probe, align the probe/target complexes, and immobilize these complexes in the nCounter cartridge. The nCounter cartridge was then placed in a digital analyzer for image acquisition and data processing. The expression level of each gene was measured by counting the number of times the color-coded barcode for that gene was detected, and the barcode counts tabulated. nSolver v4.0, an integrated analysis platform was used to generate appropriate data normalization as well as fold-changes, resulting ratios, and differential expression. nCounter™ v4.0 Advanced Analysis and R statistics were used to identify pathway-specific responses [52].

### 4.6. Recruitment of Human Burn Patients and Collection of Human Plasma and Multiplexed NanoString Measurement of miRNAs in Human EVs

Blood samples from burn patients admitted to the North Carolina Jaycee Burn Center and recruited into an IRB-approved repository protocol (IRB 04-1437) were collected and stored. Patients received standard of care and care was not affected by study participation. Patients were not excluded from the study based on burn size, inhalation injury, or factors including age, race, or substance use prior to injury. Patients were followed until discharge or expiration. This study utilized plasma samples collected early after injury (1–3 days) from 50 recruited patients.

EV were purified from plasma of burn patients and healthy donors. In order to interrogate the miRNA cargo of these EVs, we employed NanoString technology and the nCounter Human miRNA Panel v2 allowing for simultaneous evaluation of 800 miRNAs [21]. RNA was isolated from EVs which were disrupted with 10% TritonX. The exoRNeasy Kit Part II (QIAcube), and miRNA easy Kit were used for total mRNA isolation as per the manufacturer’s protocol; the quantity and quality of the mRNA in each sample was determined (A_260_/A_280_ and A_260_/A_230_) by a NanoDrop (ND1000). 100 ng of total EV mRNA was used for human NanoString nCounter miRNA microarray assay according to the manufacturer’s instructions. miRNAs were hybridized to probes at 65 °C for 30 h. Hybridized probes were extended and quantified using the nCounter Prep Station and Digital Analyzer. The nCounter-generated relative fluorescent intensities were analyzed using nSolver 4.0 software according to the manufacturer’s instructions.

### 4.7. Unbiased Proteomic Assessment of EVs from Mice and Humans and Using LC-MS/MS

EVs were isolated from sham-injured mice, 20% TBSA burn injury mice, healthy humans, and human burn patients. Plasma from mice was collected 72 h after injury, and plasma from human burn patients within the first 72 h of injury/hospital admission. After the final spin, EVs were resuspended in 20 mM Tris buffer (pH 7.5). The UNC Proteomics Core LFQ strategy was applied as described previously [53,54,55]. *Sample Preparation for Proteomics Analysis:* 8 M urea was added to the in-solution protein samples (~10–20 µg per replicate, *n* = 3), then reduced with 5 mM DTT for 30 min and alkylated with 15 mM iodoacetamide for 45 min. The samples were diluted to 1M urea, then digested with MS grade trypsin (Promega) at 37 °C overnight. The peptide samples were acidified to 1% TFA, then desalted using StrataX SPE cartridges (Phenomenex). The samples were dried via vacuum centrifugation, then resuspended in 0.1% formic acid for BCA colorimetric peptide quantitation assay (Pierce). *LC-MS/MS Analysis*: Samples were normalized, and 0.5 µg of each sample was analyzed by LC-MS/MS using an Easy nLC 1200 coupled to a QExactive HF (Thermo Scientific, Waltham, MA, USA). Samples were injected onto an Easy Spray PepMap C18 column (75 μm id × 25 cm, 2 μm particle size) (Thermo Scientific, Waltham, MA, USA) and separated over a 90 min period. The gradient for separation consisted of 5–32% mobile phase B at a 250 nL/min flow rate, where mobile phase A was 0.1% formic acid in water and mobile phase B consisted of 0.1% formic acid in ACN. The QExactive HF was operated in data-dependent mode where the 15 most intense precursors were selected for subsequent HCD fragmentation. Resolution for the precursor scan (*m*/*z* 350–1700) was set to 60,000 with a target value of 3 × 106 ions, 100 ms inject time. MS/MS scans resolution was set to 15,000 with a target value of 1 × 105 ions, 75 ms inject time. The normalized collision energy was set to 27% for HCD, with an isolation window of 1.6 *m*/*z*. Peptide match was set to preferred, and precursors with unknown charge or a charge state of 1 and ≥7 were excluded. The concentration of SAA1 protein was verified by ELISA by following the manufacturer’s instructions (R and D systems, DY2948).

### 4.8. Statistical Analyses

*LC-MS/MS.* The UNC Proteomics Core Facility is well versed in quantitative proteomics strategies to identify regulators [53,54,55,56]. Raw data files were processed using MaxQuant version 1.6.3.4 and searched against the reviewed human database (containing 20,245 sequences) or the reviewed mouse database (containing 16,940 sequences) Andromeda within MaxQuant [57]. Enzyme specificity was set to trypsin, up to two missed cleavage sites were allowed, carbamidomethylation of Cys was set as a fixed modification and oxidation of Met was set as a variable modification. Label-free quantification (LFQ) using razor + unique peptides was enabled. A 1% false discovery rate (FDR) was used to filter all data. Additional analysis was performed in Perseus GraphPad and DAVID bioinformatics. A minimum of 2 unique peptides per protein and >50% non-zero values across the datasets were required for quantification. Imputation of missing values based on normal distribution with width of 0.3 and downshift of 1.8, was performed. A LFQ log2 fold change ratio for each pairwise comparison was calculated. Student’s *t*-test was performed for each pairwise comparison (mouse burn_control; human burn_control) and a *p*-value < 0.05 was considered statistically significant. *Nanostring:* The proprietary analysis software for NanoString, nSolver v4.0, was used to appropriately normalize miRNA or mRNA levels and fold-changes, giving ratios and differential expression. For pathway analysis, nCounter™ v4.0 Advanced Analysis and Ingenuity Pathway Analysis package with robust R statistics were used to determine pathway-specific changes [52].

## 5. Conclusions

Plasma EVs released early after large burn injury enhance immune dysfunction after injury and could be used as a form of liquid biopsy to identify high-risk patients. EVs isolated within 3 days after injury from human burn patients were found to be enriched with SAA1 and CRP, which correlated with length of hospital stay in female patients. Future studies will investigate if removal of pro-inflammatory EVs improves outcomes in pre-clinical models of burn injury.

## Figures and Tables

**Table 1:** Differentially expressed proteins in plasma EVs following 20% TBSA burn injury in mice by LC-MS/MS. Key: LFQ-Label-free quantification.

Gene Name	LFQ Ratio	*p*-Value
Haptoglobin (Hp)	12.0	0.003
Serum amyloid A-1 protein (Saa1)	9.7	0.001
Myosin-4 (Myh4)	7.0	0.005
Myosin light chain 1/3 (Myl1)	6.3	0.023
Glycogen phosphorylase (Pygm)	5.6	0.000
Alpha-1-acid glycoprotein 2(Orm2)	5.0	0.014
Serum amyloid A-2 protein (Saa2)	4.8	0.039
Lysophospholipid acyltransferase 5 (Lpcat3)	3.5	0.047
Serum amyloid P-component (Apcs)	3.0	0.000
Complement C1s-B subcomponent (C1sb)	3.0	0.003
Serine protease inhibitor A3N (Serpina3n)	2.5	0.012
Alpha-1-acid glycoprotein 1 (Orm1)	2.1	0.007
Inter-alpha-trypsin inhibitor heavy chain H3 (Itih3)	2.0	0.011
C4b-binding protein (C4bpa)	1.8	0.022
Pyruvate kinase (Pkm)	1.1	0.018
Integrin alpha-IIb (Itga2b)	−1.0	0.032
Platelet glycoprotein Ib beta chain (Gp1bb)	−1.1	0.009
Gelsolin (Gsn)	−1.1	0.011
Integrin-linked protein kinase (Ilk)	−1.2	0.023
Cell division control protein 42 homolog (Cdc42)	−1.2	0.030
Regulator of G-protein signaling 10 (Rgs10)	−1.2	0.037
Beta-parvin (Parvb)	−1.2	0.035
Platelet glycoprotein Ib alpha chain (Gp1ba)	−1.2	0.044
Lymphocyte antigen 6C1 (Ly6c1)	−1.2	0.041
Chloride intracellular channel protein 1 (Clic1)	−1.3	0.048
T-complex protein 1 subunit beta (Cct2)	−1.3	0.049
Integrin beta-1 (Itgb1)	−1.3	0.028
Barrier-to-autointegration factor (Banf1)	−1.4	0.008
MOUSE 14–3-3 protein (Ywhah)	−1.5	0.011
Translocon-associated protein subunit delta (Ssr4)	−1.6	0.027
Syntaxin-11 (Stx11)	−1.7	0.045
Cysteine and glycine-rich protein 1 (Csrp1)	−1.7	0.043
Calpain small subunit 1 (Capns1)	−1.9	0.036
EGF-containing fibulin-like extracellular matrix protein 1 (Efemp1)	−1.9	0.016
Phospholipid transfer protein (Pltp)	−2.0	0.009
Ras-related protein (Rab21)	−2.0	0.039
Leukemia inhibitory factor receptor (Lifr)	−2.3	0.011
T-complex protein 1 subunit gamma (Cct3)	−2.4	0.037
Retinol dehydrogenase 11 (Rdh11)	−2.5	0.021
C-type lectin domain family 11 member A (Clec11a)	−2.6	0.035
Transmembrane protein 43 (Tmem43)	−2.6	0.021
Choline transporter-like protein 1 (Slc44a1)	−2.8	0.044
Ig kappa chain V-V region (1 SV)	−2.9	0.025
Calpain-2 catalytic subunit (Capn2)	−3.1	0.049
Synaptotagmin-like protein 4 (Sytl4)	−3.1	0.014
Protein kinase C alpha type (Prkca)	−3.4	0.028
Cytoplasmic FMR1-interacting protein 1 (Cyfip1)	−3.5	0.030
Tripeptidyl-peptidase 2 (Tpp2)	−3.7	0.002
Integrin alpha-L (Itgal)	−3.8	0.030
Vomeromodulin (Bpifb9a)	−3.8	0.005
Signal peptidase complex subunit 2 (Spcs2)	−3.9	0.006
Dimethylaniline monooxygenase (Fmo5)	−4.4	0.000
Sodium/potassium-transporting ATPase subunit alpha-2 (Atp1a2)	−4.4	0.007

**Table 2:** Differentially expressed proteins in plasma EVs from human burn patients during the first 72 h of injury by LC-MS/MS. *n* = 3 burn-injured patients, 3 healthy controls. Key: LFQ-Label-free quantification.

Gene Name	LFQ Ratio	*p*-Value
C-reactive Protein (CRP)	7.2	0.0030
Serum amyloid A-1 (SAA1)	5.9	0.0349
Lactotransferrin (LTF)	5.8	0.0212
HLA class I histocompatibility antigen (HLA-B)	5.3	0.0078
Immunoglobulin lambda constant 6 (IGLC6)	5.3	0.0488
CD59 glycoprotein (CD59)	4.9	0.0388
von Willebrand factor (VWF)	4.8	0.0306
C5a anaphylatoxin chemotactic receptor 1 (C5AR1)	4.8	0.0194
Fatty acid-binding protein, epidermal (FABP5)	4.3	0.0093
Proteoglycan 4 (PRG4)	2.8	0.0387
Monocyte differentiation antigen CD14 (CD14)	2.7	0.0160
Alpha-enolase (ENO1)	2.4	0.0494
Reelin (RELN)	2.2	0.0211
Coagulation factor V (F5)	2.2	0.0075
Fibulin-1 (FBLN1)	1.7	0.0410
CD44 antigen (CD44)	1.7	0.0201
Alpha-2-macroglobulin (A2M)	−1.0	0.0246
Alpha-2-HS-glycoprotein (AHSG)	−1.1	0.0000
Immunoglobulin lambda-like polypeptide (IGLL5)	−1.5	0.0300
Immunoglobulin heavy constant gamma (IGHG3)	−1.7	0.0091
Fetuin-B (FETUB)	−2.6	0.0340
Actin, alpha skeletal muscle (ACTA1)	−3.2	0.0348
Immunoglobulin heavy variable 3–72 OS (IGHV3-72)	−3.3	0.0355

**Table 3:** Clinical features of human burn injury patients. Key: TBSA-Total body surface area; LOS-Length of Stay; Mean ± SEM.

%TBSA Injury	Age	Sex # (%)	LOS	Mortality # (%)
18.4 ± 1.7	50.3 ±2.2	M—34 (68) F—16 (32)	49.4 ± 5.7	Overall—4 (8) M—2 (5.8) F—2 (12.5)

## Data Availability

The mass spectrometry proteomics data have been deposited to the ProteomeXchange Consortium via the PRIDE partner repository with the dataset identifier PXD028515. NanoString miRNA data has been uploaded into GEO. Other primary data can be made available from the authors upon reasonable request.

## Supplementary Materials

The following are available online at https://www.mdpi.com/article/10.3390/ijms221810083/s1.
