# Supplementary material for: Burn Injury Induces Proinflammatory Plasma Extracellular Vesicles That Associate with Length of Hospital Stay in Women: CRP and SAA1 as Potential Prognostic Indicators
Source: Int J Mol Sci. 2021 Sep 18;22(18):10083. doi: 10.3390/ijms221810083 (PMC8468249; doi:10.3390/ijms221810083)
Supplement: Supplementary file 1 [file ijms-22-10083-s001.zip › Maile et al_Table S1.pdf]

Table S1: Selected known roles of miRNAs that were altered in human burn patient EVs

**Upregulated miRNAs**	**Known roles and targets with references**
miR-663a	Enhances proinflammatory NF-kB activation[62] and inhibits TGF-B production[63]
miR-363	Promotes expression of endothelial angiocrine factors[64], and S100A1 pathway [65]
miR-4435	Secreted from UQCRB expressing lines and increased in colon cancer[66]
miR519e	Targets S100A4 pathway [67]
**Downregulated miRNAs**	Known roles
miR-505	Directly inhibits HMGB1 expression[68, 69]
miR-671	Directly targets and inhibits TLR4 expression [70]
miR-151b	Regulates osteoblast differentiation[71] and COL1A1[72]
miR-577	Inhibits HMGB1[72], and targets CXCL5[73] and CXCL9[74]
miR-431	Promotes apoptosis (33305412) and inhbits insuling signaling cascade genes[75]
miR-409	Predicted to inhibit CRP 3'UTR (TargetScan) and regulates insulin secretion from pancreas[76]
miR-1224	Inhibits Sp1-mediated cytokine secretion from NK cells [77]
miR-575	Targets PTEN [78], suppresses angiogenesis by targetting Rab5 [79]
miR-4425	Targets MTA3 [80]
miR-320b	Inhibits IFNGR [81], and inhibits angiogeneis through IGF2BP3 [82], [83]
miR-202	Predicted to inhibit CRP 3'UTR (TargetScan)
miR-338,	Inhibits CRP expression[84]
miR-605	Inhibits NFkB [85] and CXCL5 [86]
miR-520f	Predicted to inhibit CRP 3'UTR (TargetScan)
miR-342	Reduces proinflammatory responses and protects against LPS-induced acute lung injury by silencing MAPK1 [87], anti-inflammatory actions by targeting mTORC2 in Tregs [88]
miR-651	Targets Yin Yang 1 in endothelial cells [89]
miR-1180	Promotes NfkB activation through targetting NKIRAS2 [90], [91]
miR-211	Reduces inflammation by inhibiting MAPK1 **[92]), ATF6 [93], and PI3K** [94]
let-7d	Predicted to inhibit CRP 3'UTR (TargetScan)
miR-1910	Activates NFkB [95]
miR-18b	Inhibits IGF1 [96] and targets Notch2 [97]
miR-1197	Targets apoptotic factor MADD [98]
